# Impact Of Emotion Dysregulation On Eating Behavior Among The Tunisian General Population

**DOI:** 10.1192/j.eurpsy.2024.259

**Published:** 2024-08-27

**Authors:** A. Hadj Ali, M. Turki, A. Zribi, N. Halouani, S. Ellouze, J. Aloulou

**Affiliations:** ^1^Psychiatry “B” department, Hedi Chaker University Hospital, Sfax, Tunisia

## Abstract

**Introduction:**

Previous theoretical models and reviews have documented a strong connection between emotion dysregulation and eating disorders (ED) psychopathology among the general and clinical populations.

**Objectives:**

We aimed to assess the link between emotional dysregulation and ED in the Tunisian general population.

**Methods:**

We conducted a cross-sectional, descriptive and analytical study among Facebook group members, using an online questionnaire, over the period from February 17, 2023 to May 26, 2023. Emotional dysregulation was assessed via the “Difficulties in Emotion Regulation Scale” (DERS), which is composed of six sub-scores : “Non-acceptance” (N), “Strategies” (S), “Impulse” (I), “Goal” (G), “Clarity” (C) and “Awareness” (A). The Eating Attitude Test (EAT-26) was used to assess the risk of developing ED.

**Results:**

A total of 528 responses were included. The mean EAT-26 score was 12.36±10.34; and 12.3% of our population were at high risk of developing an ED. The mean N, S, I, B, Cl, C and overall DERS scores were 7.78; 8.24; 7.08; 9.57; 6.46; 7.61 and 46.74, respectively.

We showed that the EAT-26 score was correlated with the overall DERS score (r=0.260; p<0.001) as well as with the N (r=0.208; p=0.002), S (r=0.228; p<0.001), I (r=0.212; p=0,025), B (r=0.198; p<0.001), C (r=0,122; p=0,005) and Cl (r=0.136; p=0.002) scores.

**Conclusions:**

Our study showed that participants with a high risk of developing an ED seem to have more difficulties with emotional regulation. Thus, our findings call for interventions that target emotion regulation in the treatment of ED.

**Disclosure of Interest:**

None Declared

